# Temporal Dynamics of Innate Immune Activation and Viral Interference During Sequential Co-Infection with Influenza A Virus and SARS-CoV-2: Molecular Mechanisms, Clinical Evidence, and Therapeutic Implications

**DOI:** 10.3390/ijms27135994

**Published:** 2026-07-03

**Authors:** Jaime Angamarca-Iguago, Juan Marcos Parise-Vasco, Claudia Reytor-González, Jaen Cagua-Ordoñez, Daniel Simancas-Racines

**Affiliations:** Facultad de Ciencias de la Salud y Bienestar Humano, Universidad Tecnológica Indoamérica, Ambato 180150, Ecuador

**Keywords:** viral interference, innate immunity, type I/III interferons, interferon-stimulated genes, influenza A virus, SARS-CoV-2, co-infection, air–liquid interface, ORF6, IFITM3, OAS1, pegylated interferon lambda, oseltamivir, respiratory epithelium, temporal dynamics

## Abstract

The concurrent circulation of influenza A virus (IAV) and severe acute respiratory syndrome coronavirus 2 (SARS-CoV-2) has unveiled complex host–pathogen interactions governed by temporal dynamics of innate immune activation. This narrative review synthesizes evidence from human air–liquid interface (ALI) epithelial models, animal studies (hamster, ferret), clinical cohorts, and randomized controlled trials (2015–2026) to delineate the molecular mechanisms underlying viral interference between these two major respiratory pathogens. Prior IAV infection induces a robust type I/III interferon (IFN) response and broad interferon-stimulated gene (ISG) upregulation that restricts subsequent SARS-CoV-2 replication within a critical 24–72 h temporal window. Conversely, SARS-CoV-2 employs a multi-layered immune evasion strategy that blunts IFN induction, providing minimal heterologous protection. Simultaneous co-infection tends to exacerbate disease severity. Host genetic determinants, including OAS1 and TLR7 variants, modulate interference capacity. Therapeutically, early pegylated IFN-λ shows clinical benefit, while experimental evidence from in vitro and animal models suggests oseltamivir may paradoxically reduce IAV-induced interference. These findings underscore the need for multi-pathogen diagnostics, temporally informed clinical decision-making, and IFN-based therapeutic strategies during co-circulation periods.

## 1. Introduction

The emergence of severe acute respiratory syndrome coronavirus 2 (SARS-CoV-2) in late 2019 precipitated a global health crisis of unprecedented scale, fundamentally altering the epidemiological landscape of respiratory infections [1,2]. As the COVID-19 pandemic unfolded, epidemiologists observed a striking and unexpected phenomenon: the near-disappearance of seasonal influenza A virus (IAV) circulation during the 2020–2022 respiratory seasons [3,4]. While non-pharmaceutical interventions (NPIs)—including masking, social distancing, and enhanced hygiene—undoubtedly contributed to this decline, the magnitude of the effect suggested the involvement of a biological mechanism known as viral interference [5,6]. Viral interference describes a process whereby infection by one virus transiently modifies the host cellular environment, rendering it less permissive to subsequent infection by a second, heterologous virus. This concept, rooted in seminal virological observations from the 1930s and 1940s, has gained renewed prominence as a critical factor shaping the dynamics of respiratory virus co-circulation [7,8,9].

The interplay between IAV and SARS-CoV-2, two major respiratory pathogens with pandemic potential, represents a compelling case study for understanding the molecular underpinnings of heterologous viral interference. The respiratory epithelium serves as the primary portal of entry and principal replication site for both viruses [10,11]. These specialized epithelial cells form a critical barrier endowed with a sophisticated innate immune system designed to detect and respond to invading pathogens. The cornerstone of this defense is the rapid production of interferons (IFNs), a family of cytokines that orchestrate a powerful antiviral state through the induction of hundreds of interferon-stimulated genes (ISGs) [12,13,14]. The antiviral state established by IFNs is broad-acting and not specific to the inducing virus, forming the mechanistic basis for heterologous viral interference.

A pre-existing IAV infection, recognized as a potent inducer of the IFN response, could theoretically render the respiratory tract temporarily refractory to a subsequent SARS-CoV-2 challenge. However, the interaction is far from simple. The outcome of a sequential infection is critically dependent on temporal dynamics, including the precise timing, order, and interval of viral exposure [15]. The protective antiviral state induced by IAV is transient, and its efficacy against a secondary invader is contingent upon the window between the two infections. Furthermore, both IAV and SARS-CoV-2 have evolved formidable arsenals of protein antagonists that actively suppress and evade the host’s innate immune defenses, particularly the IFN pathway [10,16,17,18].

SARS-CoV-2, in particular, has been characterized as a “stealth” virus, distinguished by its remarkable ability to delay and suppress the early IFN response—a trait linked to its high transmissibility and pathogenic potential [10,19,20]. This raises a crucial question: can the robust but delayed IFN response to IAV effectively counter a virus that is exceptionally skilled at dismantling the very same pathway? Adding complexity, the emergence of SARS-CoV-2 variants of concern, particularly Omicron and its sublineages, has introduced differential IFN sensitivity profiles that may alter viral interference dynamics [21,22,23].

This narrative review aims to provide a comprehensive and mechanistically detailed synthesis of the current understanding of sequential co-infection with low pathogenic IAV and SARS-CoV-2. We present updated evidence from 2015–2026, integrating human ALI studies, in vivo animal models, clinical and epidemiological data, host genomic studies, and randomized controlled trials of IFN-λ therapeutics. We systematically dissect the molecular events occurring within the respiratory epithelium, beginning with the fundamental principles of viral interference and the intricate signaling networks of the innate immune system. We provide a comparative analysis of the molecular biology and immune evasion strategies of IAV and SARS-CoV-2, addressing key controversies surrounding IFITM3 and ORF6 function. By integrating evidence across multiple experimental platforms, we build a cohesive picture of this complex viral interplay. A central theme throughout this review is the critical role of temporal dynamics, exploring how the timing of infection dictates the balance between protective interference and potential disease exacerbation. Finally, we discuss the therapeutic and clinical implications, including the impact of oseltamivir on viral interference and the therapeutic window for IFN-λ treatment.

## 2. Methodology of the Review

### 2.1. Search Strategy

This narrative review follows an explicit methodological approach to ensure comprehensive coverage of the available literature. Data sources included MEDLINE/PubMed, Web of Science, Scopus, and Google Scholar (for initial screening). The temporal range encompassed January 2015 through January 2026. The following search terms (MeSH terms and title/abstract keywords) were used in combination: primary terms: “influenza A” AND “SARS-CoV-2” AND (coinfection OR interference) AND (interferon OR ISG OR ALI); secondary specific terms: IFITM3, ORF6, OAS1, interferon lambda, oseltamivir, viral interference, innate immunity, TLR7, host genetics, Omicron, variant.

### 2.2. Inclusion and Exclusion Criteria

Inclusion criteria comprised: peer-reviewed articles (research articles, reviews, meta-analyses); in vitro studies (cell lines, ALI human epithelium); in vivo studies (hamster, ferret, mouse models); clinical cohorts and epidemiological studies; randomized controlled trials; and studies with mechanistic or translational data. Exclusion criteria comprised: preprints without subsequent peer review; editorial decisions and peer review reports; theses and dissertations; non-indexed or grey literature.

### 2.3. Evidence Hierarchy

Evidence was hierarchized as follows: (1) meta-analyses and randomized controlled trials (RCTs); (2) prospective and retrospective cohort studies; (3) in vivo animal studies; (4) human ALI and in vitro studies. The complete search strategy, selection flowchart, and list of included/excluded studies with justification are provided in Table S1 and Figure S1 (Supplementary Materials).

## 3. Fundamentals of Viral Interference and the Interferonic Window

Viral interference is a phenomenon in which the replication of a “challenge” virus is inhibited by a pre-existing, established infection with an “interfering” virus [5,6]. This concept has deep historical roots: early observations in the 1930s and 1940s noted that monkeys infected with a neurotropic strain of yellow fever virus were resistant to subsequent challenge with a viscerotropic strain, and that influenza virus infection in embryonated eggs could prevent the growth of other viruses [7]. These foundational findings laid the groundwork for the discovery of interferons, identified as the soluble factor responsible for mediating this interference.

### 3.1. Mechanisms of Viral Interference

While IFNs are central, viral interference is a multifactorial process that operates through several distinct mechanisms. Intrinsic interference involves direct competition between viruses for essential host cell resources, including cell surface receptors for entry, nucleotide and amino acid pools, and translational machinery (ribosomes). Extrinsic (immune-mediated) interference represents the more dominant and broadly acting form, mediated by the host’s innate immune response. Upon infection, viral components are recognized by host pattern recognition receptors (PRRs), triggering signaling cascades that lead to the production of antiviral cytokines, most notably type I (IFN-α/β) and type III (IFN-λ) interferons [12,13,14].

### 3.2. The Interferon-Mediated Antiviral State

Heterologous immunity arises when the immune response to one pathogen provides cross-protection against a different pathogen [10]. IFNs act in autocrine and paracrine fashion, binding to their respective receptors and activating the Janus kinase–signal transducer and activator of transcription (JAK-STAT) signaling pathway. Activated STAT complexes translocate to the nucleus and bind to IFN-stimulated response elements (ISREs) in the promoter regions of hundreds of ISGs [24,25]. The collective action of ISG products establishes a powerful, multi-pronged antiviral state that can inhibit virtually every stage of the viral life cycle: IFITM proteins block viral entry by altering membrane fluidity; the OAS/RNase L system degrades viral and host RNA; PKR phosphorylates eIF2α, shutting down protein synthesis; MX proteins sequester viral nucleocapsids; TRIM family members function as E3 ubiquitin ligases targeting viral components; and ZAP targets CpG dinucleotides in viral RNA [25,26,27,28,29,30].

Type III IFNs (IFN-λ) are induced by IRF3/7 but signal through a distinct receptor (IFNLR1) that is preferentially expressed on epithelial cells, providing a more localized barrier defense with potentially less systemic inflammation [13,31,32]. This epithelial tropism of the IFN-λ response is particularly relevant in the context of respiratory virus co-infection, as it provides a targeted antiviral state at the site of primary viral encounter [33,34].

### 3.3. The Temporal Interferonic Window

**Figure 1:**
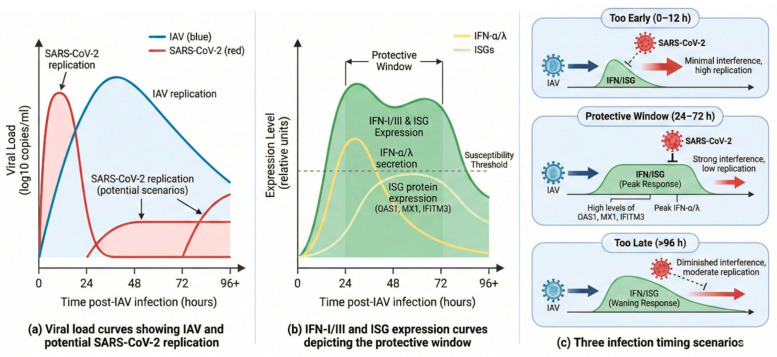
Temporal window of viral interference in sequential IAV → SARS-CoV-2 co-infection. Created in BioRender. Reytor, C. (2026) https://BioRender.com/5aygkeo (accessed on 20 June 2026). Schematic representation of the interferonic window. Panel (**a**): Viral load curves showing IAV replication (blue, peaking at 24–48 h post-infection) and potential SARS-CoV-2 replication (red) under different timing scenarios. Panel (**b**): IFN-I/III and ISG expression curves (green shaded area) depicting the protective window (24–72 h post-IAV infection). Panel (**c**): Three infection timing scenarios: Too Early (0–12 h, SARS-CoV-2 arrives before IFN peak, minimal interference); Protective Window (24–72 h, SARS-CoV-2 arrival during IFN peak, strong interference); Too Late (>96 h, IFN response waning, diminished interference). Key markers include peak IFN-α/IFN-λ secretion, ISG protein expression (OAS1, MX1, IFITM3), and susceptibility thresholds. Based on data from Cheemarla et al. [15] and Gilbert-Girard et al. [36].

Following IAV infection, the IFN signal exhibits a characteristic temporal profile: an initial delay during the first several hours post-infection, followed by an intense induction of type I and type III IFNs peaking at approximately 24–48 h, generating a “protective peak” of interference [15]. Subsequently, the response decays over days. A second virus can be effectively inhibited if it arrives within the window of 24–72 h, while very early (0–12 h) or late (>96 h) exposures lose protection. This concept of the “interferonic window” (Figure 1) is supported by both in vitro ALI data and mathematical modeling studies [5,35].

In the reverse direction, a SARS-CoV-2-first infection is expected to generate a substantially weaker and less reliable protective window against a subsequent heterologous virus such as IAV. This asymmetry reflects the molecular biology of SARS-CoV-2, a weak, delayed, and actively suppressed inducer of type I/III interferons owing to multiple viral antagonists of PRR signaling and JAK–STAT activation [10,19,20]. Consistent with this, epithelial models in which SARS-CoV-2 preceded IAV showed no significant restriction of IAV replication [36], indicating that the protective interference emphasized here is primarily a property of the robust IAV-induced IFN response rather than a reciprocal feature of both viruses.

The outcome of co-infection depends on several critical factors: (i) timing of infections, which is paramount for determining interference efficacy; (ii) order of infection, as different viruses have varying capacities to induce IFNs and evade the antiviral state; (iii) viral dose, since high doses may overwhelm the established antiviral state; and (iv) host genetic background and immune status, which modulate the strength and duration of the IFN response [15,37,38].

## 4. Molecular Mechanisms of Innate Immune Response to Respiratory Viruses

The innate immune system provides the immediate, non-specific defense against invading pathogens, and the respiratory epithelium is the key battleground where the initial interactions between host and virus occur [10,11]. Respiratory epithelial cells are equipped with a suite of germline-encoded pattern recognition receptors (PRRs) that detect conserved microbial structures known as pathogen-associated molecular patterns (PAMPs) [39].

### 4.1. Pattern Recognition Receptors

Several families of PRRs are critical for the detection of respiratory RNA viruses. Toll-like receptors (TLRs) include TLR3, which resides in endosomal compartments and recognizes double-stranded RNA (dsRNA), signaling through the TRIF adaptor to activate TBK1/IKKε and phosphorylate IRF3; and TLR7/8, which also reside in endosomes and recognize single-stranded RNA (ssRNA), signaling through MyD88 to activate IRF7 [39,40]. RIG-I-like receptors (RLRs) are cytosolic sensors: RIG-I detects short dsRNA and 5′-triphosphate RNA (a hallmark of viral genomes), while MDA5 preferentially binds long dsRNA structures. Both activate the mitochondrial antiviral-signaling protein (MAVS) upon ligand binding, leading to IRF3/IRF7 phosphorylation [41,42]. Additionally, the cGAS-STING pathway can detect cytosolic dsDNA generated during some viral infections, contributing to the IFN response [43].

### 4.2. Interferon Production and Signaling

Once phosphorylated, IRF3 and IRF7 form dimers and translocate into the nucleus, cooperating with NF-κB and AP-1 to bind promoter regions of IFN genes. The initial wave of IFN-β production establishes a powerful positive feedback loop through IFNAR signaling via the JAK1-TYK2-STAT1-STAT2-IRF9 (ISGF3) cascade. This amplification loop is critical for generating a full antiviral state within the epithelium [12,14,24].

**Figure 2:**
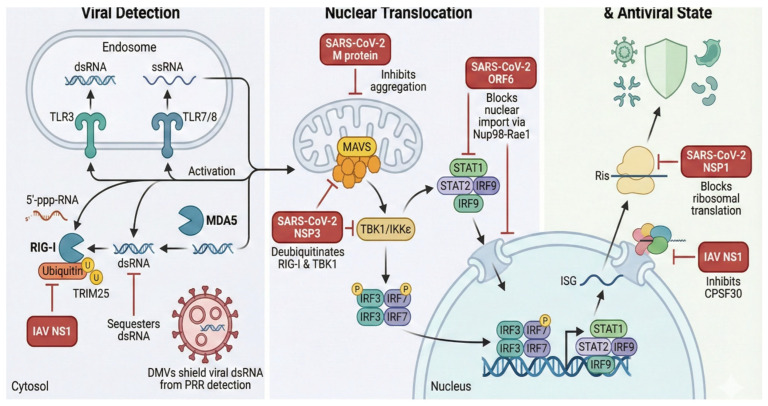
Comparative diagram of innate immune signaling and viral evasion strategies employed by IAV and SARS-CoV-2. Green arrows indicate activation pathways; red barred lines indicate viral inhibition or evasion mechanisms. Created in BioRender. Reytor, C. (2026) https://BioRender.com/5aygkeo (accessed on 20 June 2026). The diagram depicts: Left side—PRR sensing including TLR3/7/8 (endosomal) detecting viral ssRNA/dsRNA and RIG-I/MDA5 (cytosolic) detecting 5′-ppp-RNA and dsRNA. Center—signaling cascade including MAVS aggregation on mitochondria, TBK1/IKKε activation, IRF3/IRF7 phosphorylation and nuclear translocation, and STAT1/STAT2/IRF9 complex formation. Right side—ISG transcription and antiviral state establishment. Antagonism nodes (highlighted in red): IAV NS1 blocks TRIM25-RIG-I interaction, sequesters dsRNA, inhibits CPSF30; SARS-CoV-2 NSP1 blocks ribosomal translation; SARS-CoV-2 NSP3 deubiquitinates RIG-I and TBK1; SARS-CoV-2 ORF6 blocks nuclear import via Nup98-Rae1; SARS-CoV-2 M protein inhibits MAVS aggregation; SARS-CoV-2 DMVs shield viral dsRNA from PRR detection. Based on Minkoff and tenOever [10] and Kehrer et al. [44].

The host cell employs distinct primary sensing pathways and encounters unique antagonist profiles when responding to IAV versus SARS-CoV-2 (Table 1). Specifically, IAV replication intermediates and 5′-triphosphate single-stranded RNA genomes are preferentially detected in the cytosol by the RIG-I receptor, while Toll-like receptor 7 (TLR7) serves as the primary endosomal sensor for ssRNA genomes. In contrast, SARS-CoV-2, which replicates within double-membrane vesicles that shield viral double-stranded RNA from cytosolic sensors, is predominantly sensed by MDA5 recognizing long dsRNA structures, alongside TLR3/TLR7 sensing within endosomes (Table 1). The downstream IFN antagonism also differs fundamentally: IAV utilizes its NS1 protein as a master regulator to sequester dsRNA and inhibit RIG-I activation via TRIM25 binding, whereas SARS-CoV-2 deploys multiple specialized proteins, including ORF6 to block STAT1 nuclear import and NSP1 to shut down host translation. This differential reliance on distinct PRR nodes and specific molecular blockades dictates the kinetics and amplitude of the early interferon response, directly shaping the temporal dynamics of viral interference (Figure 2).

## 5. Influenza A Virus: Molecular Virulence Determinants and Innate Immune Evasion

Influenza A virus (IAV) is an enveloped virus with a segmented, negative-sense, single-stranded RNA genome belonging to the Orthomyxoviridae family. Its genome consists of eight RNA segments, each encapsidated by the viral nucleoprotein (NP) and associated with the heterotrimeric RNA-dependent RNA polymerase complex (PA, PB1, PB2), forming viral ribonucleoprotein (vRNP) complexes [45,46].

### 5.1. NS1: The Master Immune Antagonist

The primary antagonist of the host innate immune response in IAV is the non-structural protein 1 (NS1). This small, multifunctional protein acts as a master regulator of host defense evasion through multiple mechanisms: (i) sequestration of viral dsRNA via the RNA-binding domain, which binds and shields dsRNA replication intermediates, preventing detection by RIG-I, MDA5, and PKR; (ii) inhibition of TRIM25-mediated K63-linked polyubiquitination of RIG-I, essential for its activation [10,47]; (iii) inhibition of host mRNA processing through binding to CPSF30, blocking cleavage and polyadenylation of host pre-mRNAs and preventing export and translation of IFN-β and other antiviral proteins [48]; and (iv) direct interaction with PKR to inhibit its antiviral activity [16].

### 5.2. Additional IAV Immune Evasion Proteins

Beyond NS1, IAV encodes additional immune modulators. PB1-F2 localizes to mitochondria and interacts with MAVS, inducing its degradation and attenuating downstream IFN signaling [49]. PA-X, generated by ribosomal frameshifting during PA translation, possesses endonuclease activity that selectively degrades host mRNAs, thereby suppressing host gene expression including IFN and ISG transcripts [50].

### 5.3. The IAV Innate Immune Signature

Despite these evasion mechanisms, IAV antagonism is often incomplete or delayed, particularly with low pathogenic strains circulating in human populations. This results in a characteristic innate immune signature: an initial delay in IFN production during the first several hours post-infection, followed by a robust, almost explosive induction of type I and type III IFNs and a broad range of ISGs [10,15,51]. This vigorous but delayed response—termed the “delay-and-trigger” strategy—is the fundamental basis of IAV’s capacity to induce heterologous viral interference. During this process, the host cell detects IAV through endosomal and cytosolic receptors (Table 1) and initiates the transcription of numerous interferon-stimulated genes (Table 2) that collectively construct the antiviral state.

## 6. Host SARS-CoV-2 Molecular Biology and Innate Immune Interaction

Severe acute respiratory syndrome coronavirus 2 (SARS-CoV-2) is an enveloped, positive-sense, single-stranded RNA virus of the Coronaviridae family with a genome of approximately 30 kilobases encoding at least 16 non-structural proteins (NSPs), four structural proteins (Spike, Envelope, Membrane, Nucleocapsid), and numerous accessory proteins [1,52,53,54,55,56].

### 6.1. The Suppress-and-Conceal Strategy

A defining feature of SARS-CoV-2 pathogenesis is its profound and multi-faceted antagonism of the host innate immune response [10,19]. While IAV employs a “delay-and-trigger” strategy, SARS-CoV-2 employs a more comprehensive “suppress-and-conceal” strategy, leading to a significantly blunted and delayed IFN response early in infection. This distinction has been demonstrated in comparative transcriptomic analyses, which revealed that SARS-CoV-2-infected cells show markedly reduced IFN-I/III expression compared to IAV-infected cells despite similar levels of viral replication [19,20,57].

### 6.2. Multi-Level Immune Antagonism

SARS-CoV-2 deploys immune antagonism at multiple levels of the IFN signaling cascade. At the level of PAMP sequestration and evasion: double-membrane vesicles (DMVs) shield viral dsRNA from cytosolic PRRs [58,59], and RNA modifications mediated by NSP14 and NSP16 add a 5′ cap and 2′-O-methylation to viral transcripts, mimicking host mRNA [60]. At the level of host gene expression inhibition, NSP1 inserts into the 40S ribosomal subunit mRNA entry channel, blocking host mRNA translation while permitting viral protein synthesis [61]. At the level of PRR signaling disruption, NSP3 (PLpro) functions as a deubiquitinating enzyme removing activating ubiquitin chains from RIG-I and TBK1 [62,63]; NSP6 induces autophagosome formation to engulf the TBK1 signaling complex; NSP12 inhibits IRF3 phosphorylation; NSP13 binds and inhibits TBK1 [44]; and the M protein binds MAVS and inhibits its aggregation [64], while the accessory protein ORF9b inhibits RIG-I-MAVS signaling by interrupting the ubiquitination of NEMO [65]. At the level of nuclear translocation blockade, ORF6 binds the Nup98-Rae1 complex, blocking nuclear import of STAT1 and IRF3 [44,66,67]. At the level of JAK-STAT signaling interference, NSP1, NSP6, and ORF3a interfere with STAT1 phosphorylation [68,69]. These viral evasion mechanisms target key signaling adapters and transcription factors (Table 1), thereby preventing the downstream expression of key interferon-stimulated genes (Table 2) required to control viral replication. A detailed comparison of the specific viral antagonists and their target nodes for both IAV and SARS-CoV-2 is summarized in Table 3.

## 7. Controversies in Viral Immune Evasion: Ifitm3 and Orf6

### 7.1. IFITM3: Restriction vs. Facilitation

IFITM3 (interferon-induced transmembrane protein 3) is a canonical ISG that has been extensively studied as an antiviral effector. However, its role in SARS-CoV-2 infection is complex and context-dependent. Evidence for restriction includes studies demonstrating that IFITM3 restricts SARS-CoV-2 entry through the endosomal pathway by altering membrane properties and preventing viral–endosomal membrane fusion [70], with overexpression in cell lines reducing SARS-CoV-2 infection. Conversely, evidence for facilitation has emerged from studies showing that in cells with high TMPRSS2 expression (direct plasma membrane entry), IFITM proteins may paradoxically promote SARS-CoV-2 infection [71], and that endogenous IFITM levels in certain cell types can enhance SARS-CoV-1/2 replication depending on subcellular localization [72].

The outcome is determined by several factors: entry pathway (endosomal favors restriction; direct TMPRSS2-mediated favors facilitation); IFITM3 subcellular localization (endosomal membranes versus plasma membrane); expression level (high overexpression restricts; moderate endogenous levels may facilitate); and cell type (Vero and HEK293T show restriction; Calu-3 and primary airway cells show variable effects). These findings indicate that IFITM3 should be treated as an ambivalent effector whose impact depends on cellular context, entry pathway utilization, and expression levels [70,71,72]. Importantly, host genetic variants of IFITM3, particularly rs12252-C, have been associated with increased susceptibility to both severe influenza and COVID-19 in several populations [73,74].

### 7.2. ORF6: Context-Dependent Antagonism

ORF6 is a SARS-CoV-2 accessory protein characterized as a potent antagonist of IFN signaling through its interaction with the Nup98-Rae1 complex at the nuclear pore. Evidence supporting strong antagonism includes studies showing that ORF6 blocks nuclear–cytoplasmic traffic, attenuating IFN signaling in multiple experimental systems [44], with overexpression studies demonstrating significant inhibition of STAT1 nuclear translocation. However, evidence for context-dependent effects has emerged: studies in infected respiratory cells (Calu-3) show that ORF6 effects may not fully antagonize IFN signaling during actual infection [75], and the D61L variant present in Omicron sublineages shows partial loss of function [44]. ORF6 should therefore be considered a modulator rather than an absolute inhibitor of IFN signaling. Extrapolations from overexpression systems to infection contexts should be made cautiously.

## 8. Viral Interference Between Influenza A and SARS-CoV-2: Molecular Mechanisms

The epidemiological observation of reduced influenza activity during the COVID-19 pandemic strongly suggested a biological interaction between IAV and SARS-CoV-2 [3,4,76]. Subsequent mechanistic studies have confirmed that this interaction is largely unidirectional and mediated by the host’s innate immune response, with the outcome critically dependent on the timing and order of infection.

### 8.1. Scenario 1: IAV Followed by SARS-CoV-2 (Protective Interference)

IAV infection triggers a powerful type I and type III IFN response in the respiratory epithelium, leading to widespread upregulation of hundreds of ISGs. If SARS-CoV-2 encounters this pre-established, IFN-primed environment, its replication is severely restricted from the outset [15,77]. The molecular basis for this restriction involves multiple ISG effectors acting in concert: IFITM3 inhibits SARS-CoV-2 entry through the endosomal pathway; the OAS/RNase L system degrades viral RNA, with protective OAS1 variants associated with reduced COVID-19 severity [78,79]; PKR activation causes global shutdown of protein synthesis; and MX proteins, TRIM family members, and ZAP provide additional direct restriction mechanisms [25,26,27,28,29,30]. Recent studies in human ALI cultures have demonstrated that this interference effect results in several-log reductions in SARS-CoV-2 replication [15,77,80,81]. A detailed breakdown of these antiviral effectors and their specific mechanisms of action against respiratory viruses is provided in Table 2.

### 8.2. Scenario 2: SARS-CoV-2 Followed by IAV (Minimal Interference)

SARS-CoV-2 is a master of IFN suppression. When it is the primary infecting agent, it establishes a replication niche by systematically dismantling the host’s innate immune defenses through the multi-level antagonism described in Section 6.2. Subsequent IAV infection encounters little to no pre-existing antiviral state. This can lead to unrestricted replication of both viruses and potentially synergistic pathology [15,36,82].

### 8.3. Simultaneous Co-Infection

The outcome of simultaneous co-infection is complex and stochastic, depending on which virus gains the initial foothold and the relative kinetics of IFN induction versus suppression. Clinical data on simultaneous co-infections are mixed, reflecting this complex underlying biology and patient-specific factors [83,84,85,86]. In animal models, simultaneous co-infection consistently results in enhanced disease severity compared to monoinfection, suggesting that the competing viral strategies create an immunological environment detrimental to the host [87,88].

While viral interference is primarily driven by the early, localized innate interferon response, these interactions also shape subsequent adaptive immune responses. Sequential co-infection has been shown to impair the development of protective adaptive immunity; for instance, sequential exposure to both pathogens can impair neutralizing antibody production and CD4+ T cell responses, thereby potentially compromising long-term viral clearance [89]. Conversely, the immunomodulatory effects of prior infection can influence T-cell memory; oseltamivir administration, by reducing influenza-induced inflammation, can facilitate the establishment of cross-strain protective T-cell memory, demonstrating how the innate immune environment directly modulates cellular adaptive outcomes [90]. Furthermore, the kinetics of the humoral antibody response remain closely tied to early viral replication kinetics, which are themselves dictated by the presence or absence of an interferon-mediated protective window [91,92]. Thus, the adaptive immune profile is not independent of the coinfection context but is instead sequentially sculpted by the initial innate interferon dynamics.

## 9. In Vitro Evidence: Human ALI and Epithelial Cell Culture Models

### 9.1. Primary Human ALI Models

Studies using human air–liquid interface (ALI) cultures have provided the most physiologically relevant in vitro evidence for viral interference. ALI cultures recapitulate the differentiated, pseudostratified mucociliary epithelium of the human airway, making them superior to traditional submerged cell line cultures for studying respiratory virus interactions [93,94].

Cheemarla et al. (2024) [15] demonstrated that in human nasal and bronchial ALI cultures, IAV pre-infection (24–72 h) induces a robust IFN-I/III program that strongly inhibits subsequent SARS-CoV-2 replication by several orders of magnitude. Key ISGs upregulated include MX1, OAS1, and IFITM3 [15]. Critically, this same study showed that oseltamivir treatment during IAV–SARS-CoV-2 co-infection can partially reverse the interference by diminishing the IFN signal, thereby restoring SARS-CoV-2 replication—a finding with direct clinical implications for antiviral treatment decisions during co-infection [15].

Dee et al. (2023) confirmed these findings in human bronchial epithelial ALI cultures, demonstrating that both IAV and respiratory syncytial virus (RSV) trigger a cellular response that blocks SARS-CoV-2 infection through an IFN-dependent mechanism [77]. Fage et al. (2022) showed in human nasal epithelial ALI cultures that influenza A(H1N1)pdm09 virus, but not RSV, interferes with SARS-CoV-2 replication during sequential infections, highlighting pathogen-specific differences in interference capacity [80].

### 9.2. Variant Sensitivity Studies

A comprehensive study by Gilbert-Girard et al. (2024) [36] in PLOS Pathogens examined viral interference across variants and found that H3N2 interferes with both ancestral SARS-CoV-2 and Omicron variants. SARS-CoV-2 rarely inhibits IAV replication. Notably, differential sensitivity to exogenous IFN was observed: ancestral SARS-CoV-2 is less sensitive to IFN-I, while Omicron is less sensitive to IFN-III [36]. This variant-specific IFN sensitivity has been further characterized by studies demonstrating progressive IFN resistance across SARS-CoV-2 variants of concern, with Omicron BA.1 showing markedly increased resistance to type I, II, and III IFNs [21,22,23].

### 9.3. Mechanism Validation

The causal link between IFN and interference was established through several lines of evidence: (i) recombinant IFN-α or IFN-λ treatment alone inhibited SARS-CoV-2 replication in ALI cultures; (ii) interference was lost in cells deficient in MAVS, IFNAR1, or STAT1; (iii) Vero E6 cells (IFN-deficient) showed no interference, confirming the IFN-dependent mechanism; and (iv) JAK inhibitors abolished IAV-induced interference [15,36,77,95,96,97,98,99,100]. These studies collectively demonstrate that the innate immune response, specifically the IFN-I/III signaling axis, is both necessary and sufficient for mediating IAV-induced heterologous interference against SARS-CoV-2. A summary of the key in vitro and air-liquid interface (ALI) studies on IAV–SARS-CoV-2 viral interference is presented in Table 4.

## 10. In Vivo Evidence: Animal Models

### 10.1. Hamster Studies

Syrian golden hamsters have served as the primary in vivo model for studying IAV–SARS-CoV-2 co-infection. Zhang et al. (2021) and Kinoshita et al. (2021) demonstrated that simultaneous IAV–SARS-CoV-2 co-infection causes more severe and prolonged pneumonia than either virus alone, with greater weight loss and enhanced inflammatory pathology [87,88]. When IAV pre-infection preceded SARS-CoV-2 by 24–48 h, reduced SARS-CoV-2 titers were observed in lung tissue, though histopathological benefit was variable depending on dose and interval [87]. Achdout et al. (2021) further demonstrated that prior influenza immunity, but not SARS-CoV-2 immunity, prevented lethal outcomes in co-infected hamsters, underscoring the protective role of the IAV-induced immune response [101].

### 10.2. Ferret Studies

Ferrets represent an important model for upper respiratory tract infections. Ryan et al. (2022) [102] showed that prior administration of live attenuated influenza vaccine (LAIV) in ferrets did not aggravate subsequent SARS-CoV-2 infection and actually reduced SARS-CoV-2 shedding in the upper respiratory tract. This supports the concept that vaccine-induced innate priming can provide transient heterologous protection [102]. Additional ferret studies have confirmed that sequential IAV → SARS-CoV-2 infection (1-day interval) reduces SARS-CoV-2 nasal shedding, though the model is limited by its restricted lower respiratory tract pathology [89,102].

### 10.3. Key Observations from In Vivo Studies

Several consistent observations emerge from in vivo studies: (i) the acute, IFN-driven innate response during active infection mediates heterologous interference, distinct from adaptive immune memory; (ii) timing and interval are critical determinants of protection; (iii) simultaneous co-infection consistently results in worse outcomes compared to monoinfection; (iv) prior IAV infection can reduce SARS-CoV-2 replication but does not always prevent disease; and (v) the dose of the interfering virus and the interval between infections modulate the degree of protection [87,88,89,101,102]. A summary of the key in vivo animal studies investigating IAV–SARS-CoV-2 sequential and simultaneous co-infection is provided in Table 5.

## 11. Clinical and Epidemiological Evidence

### 11.1. Population-Level Interference

The near-disappearance of seasonal influenza during the 2020–2022 seasons coincided with SARS-CoV-2 pandemic waves globally. While NPIs contributed significantly, viral interference likely played a complementary role [3,4,76]. Surveillance data from the United Kingdom, Australia, Canada, the United States, and the European region consistently documented dramatic reductions in influenza circulation [103,104,105,106,107], while detailed clinical and virological studies highlighted distinct virus-host dynamics [108,109,110,111,112,113]. The global patterns and seasonal evolution of influenza continue to dictate these population-level interactions [114,115,116,117]. Mathematical models incorporating an IFN-mediated refractory period can reproduce the observed bimodal circulation patterns of respiratory viruses [5], and ecological studies from Scotland provided population-level evidence for negative interactions between influenza and other respiratory viruses [8].

### 11.2. Individual-Level Co-Infection Outcomes

Clinical studies of individually diagnosed co-infections have yielded heterogeneous results. The ISARIC-WHO UK cohort study (Swets et al., 2022), examining over 300,000 hospitalized COVID-19 patients, found that influenza co-infection was associated with significantly increased mechanical ventilation requirement and in-hospital mortality compared to SARS-CoV-2 monoinfection [83]. This finding was corroborated by a test-negative design study from Public Health England (Stowe et al., 2021), which reported a 5.92-fold increased risk of death in co-infected individuals [84]. Conversely, the Mayo Clinic/PNAS Nexus analysis (Pawlowski et al., 2022) reported low co-infection rates and no significant mortality difference after propensity matching, although more intense symptoms were observed [85], with smaller case series and dynamic models further describing these clinical interactions [118,119].

Recent meta-analyses have further quantified these outcomes. Yan et al. (2023) pooled data from 95 studies encompassing 62,107 patients, finding a 2.92-fold increased odds of mortality in co-infected patients [120]. Alizadeh-Navaei et al. (2025) estimated a global co-infection prevalence of approximately 14% among tested patients, with co-infection associated with increased intensive care unit (ICU) admission and mechanical ventilation [121]. Cong et al. (2022) confirmed that respiratory co-infection with influenza or RSV was associated with increased clinical severity of COVID-19 [122]. Other clinical cohorts and systematic reviews have similarly highlighted the variable prevalence and severity of dual infections [123,124,125,126,127,128,129].

### 11.3. Reconciling Discrepancies

The apparent discrepancy between the protective interference observed experimentally and the worse outcomes in diagnosed co-infections can be explained by several factors: (i) timing bias—diagnosed co-infections in clinical settings likely represent simultaneous or near-simultaneous infections, or SARS-CoV-2 → IAV scenarios, where interference is absent; (ii) detection bias—successful IAV → SARS-CoV-2 interference would prevent detectable secondary infection, making the protective effect invisible in clinical surveillance; (iii) NPI confounding—variable implementation across settings affects viral circulation and co-infection opportunity; (iv) variant heterogeneity—different viral strains have varying interference capacities and IFN sensitivities [21,22,23]; and (v) host factors—underlying comorbidities and immune status modulate both viral replication and immune responses [83,84,85,120,121,122].

## 12. Host Genetics and Isg Nodes

### 12.1. OAS1 as a Causal Protective Factor

Genetic studies have provided strong evidence for the causal role of the OAS–RNase L pathway in protection against severe COVID-19. Zhou et al. (2021) identified a Neanderthal-derived OAS1 isoform (p46) that protects against severe COVID-19, with elevated OAS1 levels associated with reduced hospitalization, ventilation, and death (OR 0.54 for death/ventilation) [78]. Wickenhagen et al. (2021) demonstrated that a prenylated OAS1 isoform provides enhanced protection against SARS-CoV-2 through direct antiviral activity mediated by RNase L activation [79]. Banday et al. (2022) extended these findings by showing that genetic regulation of OAS1 nonsense-mediated decay underlies the association with COVID-19 hospitalization across patients of both European and African ancestries, confirming the broad relevance of this protective mechanism [130].

### 12.2. TLR7 Loss-of-Function Variants

van der Made et al. (2020) first reported rare TLR7 loss-of-function variants in young males with severe COVID-19 requiring mechanical ventilation, establishing TLR7 as a critical innate immune sensor for SARS-CoV-2 [131]. This finding was confirmed by Fallerini et al. (2021) in a larger nested case–control study, which identified TLR7 deleterious variants in 2.1% of severely affected males under 60 years of age [132]. Furthermore, large-scale studies have mapped the genetic architecture of critical COVID-19, including inborn errors of type I IFN immunity and autoantibodies neutralizing IFNs [133,134,135,136]. Given that TLR7 senses ssRNA in endosomal compartments and is essential for IFN-I production in response to both IAV and SARS-CoV-2, these findings have direct implications for understanding individual variation in viral interference capacity.

### 12.3. IFITM3 Polymorphisms

The IFITM3 rs12252-C variant has been associated with increased susceptibility to severe influenza in multiple populations. A recent meta-analysis by Li et al. (2022) demonstrated that this variant is also associated with COVID-19 susceptibility and severity, with an odds ratio of 1.91 [73]. The dual association with both influenza and COVID-19 severity underscores the importance of IFITM3 as a shared ISG node in the defense against both pathogens and suggests that IFITM3 variants may modulate viral interference capacity.

### 12.4. Implications for Viral Interference

Host genetic variation in ISG expression and function may significantly modulate the capacity for IAV-induced interference. Individuals with higher baseline or inducible OAS1 levels may experience more robust interference, while those carrying TLR7 loss-of-function variants or IFITM3 risk alleles may have diminished IFN responses and reduced interference capacity. These considerations highlight the potential for personalized risk stratification based on innate immune gene variants [78,79,130,131,132]. Individuals with higher baseline or inducible OAS1 levels, or pre-activated nasal innate immunity as observed in children, may experience more robust interference [137,138].

## 13. Impact of SARS-CoV-2 Variant Evolution on Viral Interference

The rapid evolution of SARS-CoV-2 has introduced significant variant-specific differences in IFN sensitivity that directly impact viral interference dynamics. Progressive IFN resistance across variants of concern has been documented by multiple groups [21,22,23].

### 13.1. Omicron IFN Resistance

Nchioua et al. (2023) demonstrated that Omicron BA.1 displays reduced replication but markedly increased resistance to type I, II, and III IFNs compared to earlier variants in Calu-3, iPSC-derived alveolar, and primary airway ALI cultures [21]. Guo et al. (2022) compared the IFN sensitivity of 17 human IFNs against ancestral through Omicron variants, demonstrating progressive IFN resistance across the variant lineage [22]. Shi et al. (2024) further showed that the Omicron spike confers enhanced infectivity and IFN resistance specifically in human nasal tissue [23]. Additionally, ex vivo cultures of the human respiratory tract have demonstrated variant-specific replication kinetics, such as the replication characteristics of the Omicron BA.2 variant in upper and lower airway tissues [139].

### 13.2. Implications for IAV-Mediated Interference

The increased IFN resistance of Omicron and its sublineages suggests that the interferonic window may be narrower or less effective against newer SARS-CoV-2 variants. Gilbert-Girard et al. (2024) found that while H3N2 still interferes with Omicron replication, the differential sensitivity to IFN-I versus IFN-III between ancestral and Omicron strains may shift the relative importance of specific ISG effectors [36]. Intriguingly, Bojkova et al. (2023) reported that Omicron infection itself induces sufficient IFN signaling to block subsequent influenza A H1N1 and H5N1 virus infection, suggesting that the Omicron–IAV interaction may differ from that of earlier variants [140].

Whether the IAV-induced interferonic window remains clinically relevant against currently circulating Omicron sublineages remains plausible but not definitively proven in vivo. The evidence suggests that viral interference is highly dependent on the specific sublineage, as newer sublineages continue to accumulate mutations that enhance interferon resistance and alter entry pathways. Experimental support for continued interference is provided by human ALI models showing that prior H3N2 infection still significantly restricts Omicron replication [36]. However, the extent to which this translates to clinical protection in the general population, particularly in individuals with varying levels of vaccine-induced or infection-acquired immunity, requires further epidemiological validation.

## 14. Therapeutic Implications and Public Health Considerations

### 14.1. Pegylated IFN-λ as Early Treatment

The TOGETHER trial (Reis et al., 2023 [141]), a multicentric Phase 3 RCT, demonstrated that a single subcutaneous dose of pegylated IFN-λ (180 μg) within 7 days of COVID-19 symptom onset significantly reduced the combined endpoint of hospitalization or emergency department visit by 51% and death by 50% in outpatients (n = 1936). The effect was greatest when administered within 3 days of symptom onset, aligning with the concept of early innate immune augmentation within the interferonic window [141].

The ILIAD trial (Feld et al., 2021), a Phase 2 placebo-controlled RCT, showed that peginterferon lambda treatment resulted in an odds ratio of 4.12 for undetectable virus by day 7 in outpatients with COVID-19 [142]. Conversely, the Stanford trial (Jagannathan et al., 2021) showed no significant difference in viral shedding, though this study had a smaller sample size and different patient population [143]. These results collectively support the therapeutic potential of IFN-λ, particularly when administered early in infection, but highlight the critical importance of timing.

Mechanistically, IFN-λ is particularly suited for respiratory virus treatment due to its preferential action on epithelial cells through the IFNLR1 receptor, providing localized antiviral defense with reduced systemic inflammation compared to IFN-I [13,31,32]. However, prolonged IFN-λ exposure may paradoxically impair epithelial repair, as demonstrated by Broggi et al. (2020), underscoring the need for precise temporal administration [144].

An additional layer of complexity in interferon-based therapies is presented by the finding that angiotensin-converting enzyme 2 (ACE2), the primary receptor for SARS-CoV-2 entry, is itself an interferon-stimulated gene [145] or contextually induced [146,147]. This dual nature of ACE2 creates a theoretical paradox wherein exogenous interferon administration, while intended to suppress viral replication, could simultaneously upregulate receptor expression and potentially facilitate viral entry. While this transcriptional induction has been observed in various experimental settings, it does not seem to negate the robust antiviral state and replication restriction demonstrated in human air-liquid interface (ALI) models, suggesting that downstream effector ISGs successfully outcompete any receptor upregulation. Nonetheless, this mechanism underscores the necessity of careful patient selection and precise temporal dosing to ensure that the protective antiviral threshold is established before viral entry dynamics can be enhanced.

### 14.2. Impact of Anti-IAV Antivirals on Interference

A critical clinical consideration is the effect of anti-IAV antivirals on viral interference. In human ALI models, oseltamivir treatment can reduce the IAV-induced IFN signal, thereby partially restoring SARS-CoV-2 replication [15]. This finding has been supported by in vivo studies: Bird et al. (2015) demonstrated that oseltamivir prophylaxis markedly reduces innate immune responses including TNF-α, IL-6, and IFN-γ in both mice and humans [90], and Takahashi et al. (2010) showed that oseltamivir attenuates inducible respiratory immune responses in IAV-infected mice [148].

These observations suggest potential clinical implications: in confirmed co-infection, both viruses should be treated with appropriate antivirals; viral and IFN dynamics should be monitored where feasible; and the timing of anti-IAV treatment may influence SARS-CoV-2 outcomes, particularly in the early phases of co-infection when interference is most active [15,90,148].

It is critical to note, however, that the finding that oseltamivir administration reduces influenza-induced interference is derived exclusively from human in vitro air–liquid interface (ALI) epithelial models [15] and rodent studies [90,148], rather than clinical cohorts. To establish this effect in a clinical setting and inform treatment protocols, robust human epidemiological evidence is needed. This would ideally involve retrospective or prospective cohort studies of co-infected patients analyzing whether clinical outcomes and SARS-CoV-2 viral load clearance kinetics differ significantly in those treated with neuraminidase inhibitors compared to untreated controls, alongside longitudinal evaluations of mucosal interferon dynamics in vivo.

### 14.3. Multi-Pathogen Diagnosis

During periods of co-circulation, multiplex RT-PCR panels for respiratory pathogens are essential to identify at-risk co-infections, guide appropriate combination therapies, and inform prognosis and management decisions [83,149]. The increasing availability and decreasing cost of multiplex molecular diagnostics have made multi-pathogen testing feasible in many clinical settings.

### 14.4. Vaccination Strategies

Influenza and COVID-19 vaccination campaigns serve dual purposes: reducing community viral loads and co-infection probability, and potentially providing transient innate priming effects. Ryan et al. (2022) demonstrated that LAIV administration in ferrets conferred transient heterologous protection against SARS-CoV-2 [102]. Combined vaccination campaigns optimize resource utilization and maximize population-level protection against both pathogens [149,150]. A comprehensive summary of therapeutic considerations, including antivirals, interferons, and vaccination strategies during co-infection, is presented in Table 6.

## 15. Limitations of Current Evidence

### 15.1. Model Limitations

ALI and in vitro models, while physiologically superior to submerged cultures, do not fully capture systemic immunity, comorbidities, or tissue architecture complexity. They lack immune cell recruitment and the complex multicellular interactions present in vivo. Animal models have species-specific differences in receptor expression, pathology, immune responses, and disease progression that limit direct extrapolation to human disease [87,88,89,102].

### 15.2. Clinical Study Limitations

NPI confounding represents a significant challenge, as variable implementation across settings and time periods affects viral circulation and co-infection opportunity. Rapid viral evolution limits the generalizability of findings across time, particularly given the progressive IFN resistance observed in newer SARS-CoV-2 variants [21,22,23]. Timing uncertainty is a major issue in clinical studies, as infection order and interval are rarely known with precision in clinical settings. Point-in-time sampling may miss sequential infections, leading to underestimation of interference events [83,84,85,120,121,122].

### 15.3. Mechanistic Uncertainty

The context-dependent effects of IFITM3 complicate therapeutic targeting strategies [70,71,72]. Variable ORF6 function across experimental systems and viral variants limits mechanistic generalization [44,75]. ISG redundancy—the fact that multiple ISG effectors contribute to antiviral defense simultaneously—makes single-target interventions complex and potentially insufficient [25,26,27,28,29,30].

## 16. Research Priorities and Future Directions

### 16.1. Immediate Priorities

Several immediate research priorities emerge from this review: (i) longitudinal studies with serial sero- and virology to infer infection order and interval in natural settings; (ii) interference mapping by variant (Omicron sublineages, H3N2/H1N1) in ALI models with pharmacological interventions (oseltamivir, IFN-λ); (iii) pharmacology of interference, including host-directed strategies such as local IFN-λ stimulation for short-term prophylaxis in high-risk exposures; and (iv) validation of oseltamivir’s effect on interference in clinical co-infection cohorts.

### 16.2. Longer-Term Goals

Longer-term research goals include: (i) host genomics integration through stratification by OAS1, TLR7, IFITM3, and other ISG variants for personalized risk assessment; (ii) combination therapeutic trials testing optimal antiviral combinations in confirmed co-infection; (iii) epidemiological modeling with integration of interference parameters into pandemic preparedness models; and (iv) pan-respiratory virus interference mapping to extend the framework beyond IAV and SARS-CoV-2 to include RSV, rhinovirus, and other respiratory pathogens.

## 17. Conclusions

The interaction between IAV and SARS-CoV-2 is governed by time: prior IAV infection can “shield” the respiratory epithelium through IFN/ISG induction and restrict SARS-CoV-2 replication within a critical 24–72 h interferonic window; simultaneous co-infection or SARS-CoV-2-first scenarios can aggravate disease. The temporal dynamics of innate immunity play a pivotal role in determining the outcome of sequential infections.

Key clinical implications include: (i) multi-pathogen testing during co-circulation periods is essential; (ii) clinicians should consider the potential impact of anti-IAV antivirals on interference when co-infection is suspected; (iii) early IFN-λ treatment may benefit appropriate candidates, particularly within the first 3 days of symptom onset; and (iv) vaccination campaigns targeting both influenza and COVID-19 reduce co-infection risk and community transmission.

For pandemic preparedness, epidemiological models should integrate viral interference parameters; public health strategies should account for heterologous viral interactions; and host genetic variation may inform personalized risk stratification. The progressive IFN resistance observed across SARS-CoV-2 variants underscores the dynamic nature of viral interference and the need for continued surveillance. While IAV-induced IFN can provide short-term heterologous protection, the clinical implications are complex and depend on a delicate balance between viral replication kinetics and host immune responses. Further research into the precise molecular determinants of viral interference will be essential for informing future pandemic preparedness and therapeutic development.

## Figures and Tables

**Table 1:** Key pattern recognition receptors (PRRs) and signaling adaptors relevant to IAV and SARS-CoV-2 detection.

PRR	Ligand	Compartment	Adaptor	Key Outputs
TLR3	dsRNA	Endosome	TRIF	IFN-I, IFN-III, pro-inflammatory cytokines
TLR7/8	ssRNA	Endosome	MyD88	IFN-I, IFN-III, pro-inflammatory cytokines
RIG-I	5′-pppRNA, short dsRNA	Cytosol	MAVS	IFN-I, IFN-III, pro-inflammatory cytokines
MDA5	Long dsRNA	Cytosol	MAVS	IFN-I, IFN-III, pro-inflammatory cytokines
cGAS	Cytosolic dsDNA	Cytosol	STING	IFN-I, pro-inflammatory cytokines

**Table 2:** Key interferon-stimulated genes (ISGs) and their antiviral mechanisms.

ISG	Function	Mechanism of Action
IFITM1/2/3	Block viral entry	Alter endosomal membrane curvature and rigidity
OAS1/2/3	RNA degradation	Synthesize 2′-5′ oligoadenylates activating RNase L
PKR	Translation inhibition	Phosphorylate eIF2α, shutting down protein synthesis
MxA/Mx1	Viral sequestration	Trap ribonucleoprotein complexes via GTPase activity
TRIM family	Ubiquitination	Target viral components for proteasomal degradation
ZAP	RNA degradation	Target CpG dinucleotides in viral RNA
BST2/Tetherin	Virion retention	Prevent budding of enveloped viruses
Viperin	Lipid raft disruption	Inhibit viral budding and replication complexes

**Table 3:** Viral antagonists of IFN pathways: IAV versus SARS-CoV-2.

Virus	Protein	Host Target	Mechanism	Model
IAV	NS1	RIG-I, TRIM25, CPSF30	Sequesters viral RNA, inhibits ubiquitination, blocks host mRNA processing	In vitro, in vivo
IAV	PB1-F2	MAVS	Mitochondrial localization, MAVS degradation	In vitro
IAV	PA-X	Host mRNAs	Endonuclease-mediated host mRNA degradation	In vitro, in vivo
SARS-CoV-2	NSP1	40S ribosome	Blocks host mRNA translation (host shutoff)	In vitro
SARS-CoV-2	NSP3 (PLpro)	IRF3, TBK1	Deubiquitination and inactivation of signaling molecules	In vitro
SARS-CoV-2	ORF6	Nup98-Rae1	Blocks nuclear import of STAT1 and IRF3 (context-dependent)	In vitro
SARS-CoV-2	M protein	MAVS	Binds and inhibits MAVS aggregation	In vitro
SARS-CoV-2	NSP13	TBK1	Binds and inhibits TBK1 phosphorylation	In vitro
SARS-CoV-2	DMVs	Cytosolic PRRs	Shield viral dsRNA from detection	In vitro
SARS-CoV-2	NSP16	MDA5	2′-O-methylation of viral RNA prevents MDA5 sensing	In vitro

**Table 4:** Summary of ALI/in vitro evidence for viral interference (2020–2025).

Model	Infection Order	Interval	Effect on SARS-CoV-2	Key Finding	Ref.
Human nasal/bronchial ALI	IAV → SARS-CoV-2	24–72 h	Strong inhibition (several logs)	Oseltamivir reverses interference	[15]
Human ALI	H3N2 → SARS-CoV-2 (ancestral/Omicron)	24–48 h	Significant reduction	H3N2 interferes with all variants tested	[36]
Human bronchial ALI	IAV/RSV → SARS-CoV-2	24–48 h	Significant reduction	IFN-dependent mechanism confirmed	[77]
Human nasal ALI	H1N1 → SARS-CoV-2	24 h	Significant reduction	H1N1 but not RSV interferes	[80]
Calu-3	IAV → SARS-CoV-2	48 h	Significant reduction	IFN-λ dominant in epithelial response	[81]
A549-ACE2	SARS-CoV-2 → IAV	12 h	No significant change in IAV	SARS-CoV-2 does not protect against IAV	[36]
Vero E6	IAV → SARS-CoV-2	24 h	No interference	Confirms IFN-dependent mechanism	[15,36]
Human ALI + oseltamivir	IAV → SARS-CoV-2 + Tx	24 h	Partial restoration	Antiviral treatment impacts interference	[15]

**Table 5:** Summary of in vivo evidence for IAV–SARS-CoV-2 co-infection (hamster/ferret studies).

Species	Design	Order/Interval	Outcomes	Key Limitations	Ref.
Syrian hamster	Simultaneous	IAV + SARS-CoV-2 (day 0)	↑ Weight loss, ↑ pneumonia severity	Single time point, one strain each	[87]
Syrian hamster	Simultaneous	IAV + SARS-CoV-2 (day 0)	More severe and prolonged pneumonia	Limited histological time points	[88]
Syrian hamster	Sequential	IAV → SARS-CoV-2 (24–48 h)	↓ SARS-CoV-2 titers; variable histopath	Dose-dependent effects	[87]
Syrian hamster	Sequential	IAV immunity → co-infection	Influenza immunity prevents lethality	Pre-existing immunity only	[101]
Ferret	Sequential (LAIV)	LAIV pre-treatment	No aggravation; ↓ SARS-CoV-2 shedding	Limited pathology assessment	[102]
Ferret	Sequential	IAV → SARS-CoV-2 (1 day)	↓ SARS-CoV-2 nasal shedding	Upper tract restricted model	[89]

**Table 6:** Therapeutic considerations in IAV–SARS-CoV-2 co-infection.

Intervention	Mechanism	Evidence Level	Key Considerations
Peg-IFN-λ (early)	Augments epithelial innate response	Phase 3 RCT (TOGETHER)	Most effective ≤ 3 days; outpatient setting
Oseltamivir/NAIs	Reduce IAV replication	Standard care	May reduce IFN signal; monitor in co-infection
Nirmatrelvir/ritonavir	Inhibits SARS-CoV-2 protease	Standard care	Use in confirmed co-infection
Multiplex PCR diagnosis	Identifies co-infection	Standard of care	Essential during co-circulation peaks
Influenza vaccination	Reduces IAV burden	Public health policy	May provide transient innate priming
COVID-19 vaccination	Reduces SARS-CoV-2 burden	Public health policy	Reduces severe co-infection risk

## Data Availability

No new data were created or analyzed in this study. Data sharing is not applicable to this article.

## Supplementary Materials

The following supporting information can be downloaded at: https://www.mdpi.com/article/10.3390/ijms27135994/s1.
