# Supplementary material for: Temporal Dynamics of Innate Immune Activation and Viral Interference During Sequential Co-Infection with Influenza A Virus and SARS-CoV-2: Molecular Mechanisms, Clinical Evidence, and Therapeutic Implications
Source: Int J Mol Sci. 2026 Jul 3;27(13):5994. doi: 10.3390/ijms27135994 (PMC13360870; doi:10.3390/ijms27135994)
Supplement: Supplementary file 1 [file ijms-27-05994-s001.zip › ijms-4308536-supplementary.pdf]

Supplementary Materials

Temporal Dynamics of Innate Immune Activation and Viral Interference during Sequential Co-Infection with Influenza A Virus and SARS-CoV-2: Molecular Mechanisms, Clinical Evidence, and Therapeutic Implications

Type of review and transparency statement

This article is a narrative (non-systematic) review. A narrative design was chosen because the question spans heterogeneous evidence (molecular/cell-biology studies, human ALI models, animal models, clinical cohorts, host genetics, and randomized trials) requiring integrative critical interpretation rather than a single quantitative synthesis. Accordingly, the review does not follow the PRISMA 2020 reporting guideline, which was developed for systematic reviews and meta-analyses and has no consensus extension for narrative reviews; the design and reporting were instead guided by methodological recommendations for narrative reviews and informed by the SANRA quality criteria. To preserve transparency, we document the search sources and strategy (Section A), the eligibility framework (Section B), the evidence hierarchy (Section C), and a simplified study-selection flow (Figure S1). The structured PubMed/MEDLINE search covered literature published from January 2015 through January 2026 (consistent with the Methods) and is fully reproducible using the strings in Section A with a publication-date limit of January 2026, yielding 20 records (9 excluded at screening; 11 eligible from that database arm). Combined with the other databases (Web of Science, Scopus, Google Scholar) and reference-list / citation searching, the synthesis incorporated 22 primary empirical studies that directly address IAV–SARS-CoV-2 interference / co-infection (see Section D and Figure S1). Consistent with a narrative review, per-stage counts for the supplementary routes were not prospectively logged and are documented as search strategies rather than reconstructed as numbers. The manuscript additionally cites supporting mechanistic and background literature that is not part of the study-selection flow.

Table S1. Search strategy, eligibility criteria, and evidence hierarchy.

Section A. Database-specific search strategy

MEDLINE/PubMed — primary (core) string [literature published Jan 2015–Jan 2026 → 20 records; reproducible]: ("Influenza A virus"[MeSH] OR "influenza A"[tiab] OR IAV[tiab]) AND ("SARS-CoV-2"[MeSH] OR "SARS-CoV-2"[tiab] OR "COVID-19"[MeSH]) AND (coinfection[tiab] OR co-infection[tiab] OR interference[tiab] OR "viral interference"[tiab]) AND (interferon[tiab] OR interferons[MeSH] OR ISG[tiab] OR "interferon-stimulated gene"[tiab] OR ALI[tiab] OR "air-liquid interface"[tiab]). Filter: 2015/01/01–2026/01/31.

MEDLINE/PubMed — secondary targeted terms (adjunct searches, each combined with the primary pathogens): IFITM3; ORF6; OAS1; "interferon lambda"/"IFN-lambda"; oseltamivir; "viral interference"; "innate immunity"; TLR7; "host genetics"; Omicron; variant.

Web of Science (Core Collection): TS=(("influenza A" OR "influenza A virus" OR IAV) AND ("SARS-CoV-2" OR COVID-19) AND (coinfection OR "co-infection" OR interference OR "viral interference") AND

(interferon OR ISG OR "interferon-stimulated gene" OR ALI OR "air-liquid interface")); secondary terms as above. Timespan 2015–2026; document types: article, review, meta-analysis, clinical trial.

Scopus: TITLE-ABS-KEY(("influenza A" OR "influenza A virus" OR IAV) AND ("SARS-CoV-2" OR COVID-19) AND (coinfection OR "co-infection" OR interference OR "viral interference") AND (interferon OR ISG OR "interferon-stimulated gene" OR ALI OR "air-liquid interface")); secondary terms as above. Publication years 2015–2026; document types: article, review, meta-analysis.

Google Scholar: "influenza A" "SARS-CoV-2" (coinfection OR "co-infection" OR interference) (interferon OR ISG OR "air-liquid interface" OR ALI); custom range 2015–2026; used for initial screening and supplementary citation checking only.

Per-database record counts other than the PubMed primary anchor were not prospectively logged; consistent with a narrative review, only the documented search strings are reported.

Section B. Eligibility criteria and justification

Criterion	Justification
INCLUSION — Peer-reviewed articles (research, reviews, meta-analyses)	Evidence base must be peer-validated and citable.
INCLUSION — In vitro studies (cell lines, ALI human epithelium)	Directly inform epithelial and interferon-mediated interference mechanisms.
INCLUSION — In vivo studies (hamster, ferret, mouse)	Temporally controlled sequential-infection evidence not obtainable in humans.
INCLUSION — Clinical cohorts and epidemiological studies	Link mechanism to co-circulation, prevalence, and disease severity.
INCLUSION — Randomized controlled trials	Inform therapeutic implications, particularly interferon-based interventions.
INCLUSION — Studies with mechanistic or translational data	Match the review objective (mechanisms to clinical to therapeutic).
EXCLUSION — Preprints without subsequent peer review	Avoid reliance on non-validated findings.
EXCLUSION — Editorial decisions and peer-review reports	Not primary mechanistic/clinical/epidemiological evidence.
EXCLUSION — Theses and dissertations	Not uniformly indexed or peer-reviewed.
EXCLUSION — Non-indexed or grey literature	Maintain a reproducible indexed evidence base.

Section C. Evidence hierarchy

(1) meta-analyses and randomized controlled trials (RCTs); (2) prospective and retrospective cohort studies; (3) in vivo animal studies; (4) human ALI and in vitro studies.

Section D. Included evidence accounting

Primary empirical studies included as direct evidence on IAV–SARS-CoV-2 interference / co-infection: n = 22 — in vitro / ALI (Table 4): 5 (refs 15, 71, 74, 75, 76); in vivo (Table 5): 5 (refs 82, 83, 87, 88, 89); clinical, individual-level (cohorts and meta-analyses): 6 (refs 78, 79, 80, 96, 97, 98); population / ecological (ecological and surveillance reports): 6 (refs 8, 90, 91, 92, 93, 94). The structured PubMed search (Figure S1) contributed 11 of these studies; the remainder were identified through the other databases and reference-list / citation searching. Mathematical-modelling, contextual (non-pharmaceutical-intervention), and variant-biology references, and the broader

mechanistic/background literature (approximately 150 references cited in total, pending reference management), constitute supporting literature and are not part of the study-selection flow.

Figure S1. Simplified study-selection flow (narrative review).

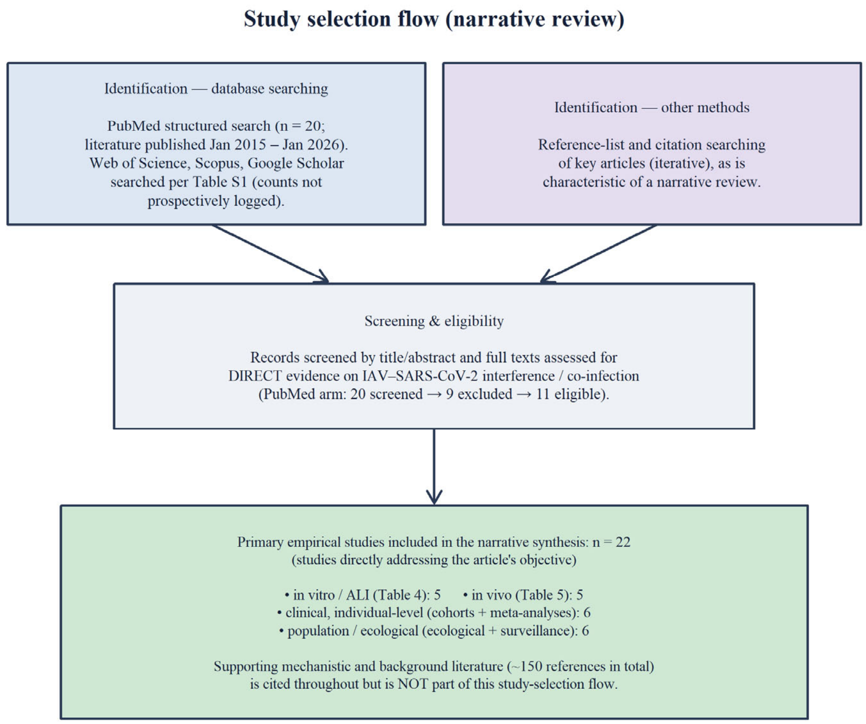

Figure S1. Simplified flow of study identification and selection for this narrative review, depicting only the primary empirical studies that provide direct evidence on IAV–SARS-CoV-2 interference / co-infection (n = 22). Two identification routes are shown: structured database searching (PubMed search covering literature published January 2015–January 2026, with reproducible counts; Web of Science, Scopus, and Google Scholar searched per Table S1) and reference-list / citation searching. The diagram is a transparency aid inspired by PRISMA principles and is not a formal PRISMA 2020 systematic-review flow diagram. Supporting mechanistic and background literature cited in the manuscript is not represented in this selection flow.
